# Effects of L-dopa on expression of prolactin and synaptotagmin IV in 17β-estradiol-induced prolactinomas of ovariectomized hemiparkinsonian rats

**DOI:** 10.17305/bjbms.2021.5491

**Published:** 2021-12

**Authors:** Maja Zorović, Kaja Kolmančič, Marko Živin

**Affiliations:** 1 Brain Research Laboratory, Institute of Pathophysiology, Medical Faculty, University of Ljubljana, Slovenia; 2 Clinical Department of Nuclear Medicine, Ljubljana University Medical Centre, Ljubljana, Slovenia

**Keywords:** Parkinson’s disease, synaptotagmin IV, L-dopa, 17b-estradiol, prolactin, prolactinoma

## Abstract

Parkinson’s disease (PD) is a long-term degenerative disorder of the central nervous system that mainly affects the motor system. Dopamine precursor levodopa (L-dopa) is used as the first-line treatment for PD. Evidence suggests neuroprotective effects of estrogens in PD. Since both 17b-estradiol (E2) and L-dopa act as regulators of prolactin (PRL) secretion from the pituitary gland, we investigated their effect on the expression of PRL in prolactinomas that developed in ovariectomized hemiparkinsonian rats treated with E2. We also investigated the effect of E2 and L-dopa on the expression of synaptotagmin IV (Syt IV), an immediate early gene whose product is abundant in the pituitary gland and was found to be highly co-expressed with PRL in lactotrophs (>90%). The hemiparkinsonian rat model was obtained by unilateral lesioning of dopaminergic nigrostriatal neurons. Rats received silastic tubing implants with E2 and were treated with L-dopa. Enzyme-linked immunosorbent assay and immunohistochemistry were used to assess the serum concentrations of PRL and E2 and expression of PRL and Syt IV in the tissue of adenohypophysis, respectively. We found that high levels of serum E2 were associated with the upregulation of Syt IV and PRL in PRL-ir cells, while treatment with L-dopa decreased the size of prolactinomas and downregulated Syt IV but had no effect on PRL expression or serum concentrations.

## INTRODUCTION

Parkinson’s disease (PD) is a neurodegenerative disorder characterized by motor and non-motor symptoms and a second most common neurodegenerative disorder after Alzheimer’s disease [1]. The major pathologic feature of PD is a progressive loss of dopamine-producing neurons of the substantia nigra (SN), resulting in a reduction of the dopamine content in the target field of these neurons, the striatum [2]. Dopamine precursor levodopa (L-dopa) is the most common first-line treatment for motor symptoms of PD. Increasing evidence suggests that estrogens may protect motor neurons and reduce the motor symptoms of PD [3]. Epidemiological studies associate post-menopausal estrogen treatment with a reduction in risk of PD [4]. Injections of submaximal doses of 6-hydroxyd induces a lesion in the nigrostriatal dopaminergic pathway similar to that seen in PD, showed sexual dimorphic susceptibility in rats [5,6]. In females, endogenously produced estrogen acted neuroprotectively and reduced dopamine depletion compared to males. Treatment with 17b-estradiol (E2) resulted in significant amelioration of the behavioral and biochemical alterations induced by 6-OHDA in female rats [7]. Dopamine and estrogen are major opposing regulators of the endocrine functions of pituitary lactotrophs, specialized cells of the anterior pituitary gland which synthesize and secrete a polypeptide hormone prolactin (PRL) [8,9]. Estrogen treatment stimulates the secretion of PRL and plays a role in development of PRL-secreting pituitary tumors called prolactinomas [10,11]. In the hypothalamic-pituitary-PRL axis, dopamine holds a predominant role in the regulation of PRL secretion. It binds to D2 receptors expressed on the cell membrane of the lactotrophs. D2 receptors are negatively linked to the adenylate cyclase system and their activation results in a reduction of PRL exocytosis and gene expression [12]. In the treatment of hyperprolactinemia due to prolactinomas, dopamine agonists such as cabergoline or bromocriptine are well established as the first line treatments.

Secretion of hormones from endocrine cells as well as release of neurotransmitters from neurons are two processes that both rely on calcium (Ca^2+^) ions entering through voltage-gated channels and Ca^2+^-dependent fusion of secretory vesicles with the plasma membrane. Synaptotagmin IV (Syt IV) is a transmembrane protein located in the Golgi apparatus, synaptic vesicles, and/or dense core vesicles with a multifunctional role in Ca^2+^-dependent exocytosis [13]. Syt IV is also an immediate early gene, which can be induced by depolarization in PC12 cells and in the hippocampus. In the hemiparkinsonian rats unilateral lesions of dopaminergic nigrostriatal neurons resulted in transient upregulation of Syt IV mRNA within the dopaminergically hypersensitive striatum through the stimulation of striatal dopamine D1 receptor with L-dopa through adenylate cyclase activity [14,15]. Previous studies have shown that Syt IV can be either a negative or a positive regulator of Ca^2+^-dependent exocytosis, suggesting a multi-functional role of Syt IV [13]. Syt IV also plays an important role in neurodegenerative processes [16]. It has been shown that pituitary gland has much higher levels of Syt IV than the brain which suggests a role in neurosecretion [17].

The aim of our study was to investigate the changes in expression of Syt IV and PRL in pituitary glands of hemiparkinsonian rats during treatment with L-dopa. Since both PRL and dopamine levels are tightly linked to estrogen, we wanted to avoid the fluctuations in estrogen levels that occur during the normal estrous cycle of the rat [18,19]. This was achieved by performing ovariectomy on all experimental animals submaximal doses of 6-hydroxydopamine (6-OHDA), which subcutaneous silastic capsules [20,21]. In accordance with studies described in the literature [21,22], we found that in all E2-treated animals, higher than physiological serum levels of E2 resulted in pituitary hyperplasia.

Due to its specific structure and function among synaptotagmins, Syt IV has received a lot of attention over the past two decades [13-17,23], and our investigation advances that knowledge by providing data on effects of L-dopa on Syt IV expression in pituitary glands of postmenopausal hemiparkinsonian rats with estrogen implants, with relevance for female PD patients on estrogen hormonal replacement therapy.

## MATERIALS AND METHODS

### Animals

Female Wistar rats weighing 200-300 g were obtained from the Medical Experimental Center, Medical Faculty, Ljubljana, Slovenia. A total of 15 rats were used in the study. Rats were housed in polycarbonate cages (Ehret IV, Mahlberg, Germany, floor area 1825 cm^2^) with sterilized bedding material (Lignocel ¾ and Rehofix, JRS, Germany) and sterilized cellulose towels as nesting material in a temperature-controlled colony room at 22-24°C (relative humidity 35-60%) under a 12 h light/12 h dark cycle with free access to autoclaved water and maintenance rodent diet (1320 Altromin, Lage, Germany). Following ovariectomy, standard pellet food was replaced by a low-phytoestrogen diet (Teklad Global 14% Protein Rodent diet 2014, Envigo, Huntington, UK). Animals were treated according to the Directive 2010/63/EU of the European Union and the National Veterinary Institute Guide for the Care and Use of Laboratory Animals. The protocol was approved by the Veterinary Administration of the Republic of Slovenia (Protocol Number U34401-7/2017/9). Every effort was made to minimize the suffering and the number of animals used in this study. Before and after the 6-OHDA, implant, and ovariectomy surgeries, analgesic meloxicam (Meloxidyl, Accord Healthcare Ltd., Middlesex, UK) was administered to each animal. Animal health and wellbeing were monitored daily throughout the study and hourly following the surgeries. A scoring system was put in place for close monitoring of clinical signs (hydration, breathing, body weight [BW], locomotion, the appearance of fur, and discharge from the eyes or nose) and for establishing humane endpoints following the guidelines outlined in Directive 2010/63/EU of the European Union. No animals reached the criteria for applying humane endpoints and no mortality occurred outside of planned euthanasia.

### Design of the study

At the beginning of the study, ovariectomy was performed on 10 animals, which were then divided into two groups. An additional group of 5 intact healthy female rats was included in the study to allow comparison with the “normal” condition. Two weeks following ovariectomy, 6-OHDA lesioning surgery was performed on animals in the first two groups and at the same time E2 filled silastic tubing depos were implanted at the nape of the neck in the same animals. Two weeks following the 6-OHDA lesioning and implant surgeries, daily injections of L-dopa or saline were commenced. After another 2-week period, the animals were euthanized with CO_2_ (3 days after the end of daily injections). Afterward, they were decapitated and the whole brains together with pituitary glands were rapidly removed and frozen on dry ice and kept at −20°C until they were sectioned in a cryostat at -20°C (Leica CM1950, Leica Biosystems, Wetzlar, Germany). The tissue was cut into 10 mm coronal sections. Sections were mounted to microscope glass slides coated with 0.01% solution of poly-L-lysine (Sigma). Slides with tissue sections were then vacuum packed and stored in a freezer at −20°C until processed for immunohistochemistry. Blood was collected from the chest cavity and/or from the heart into centrifuge tubes and after being kept at room temperature for 20 minutes, it was centrifuged 10 minutes at 3000 rpm. The serum was aliquoted and kept at −20°C until further processing.

### Unilateral 6-OHDA lesions of nigrostriatal pathway

Animals were anesthetized by i.p. injection of 2 ml/kg of body weight (BW) anesthetic (mixture of 2% xylazine hydrochloride (Chanazine, Chanelle Pharmaceuticals Manufacturing Limited, Galway, Ireland; 8 mg/g BW) and 10% ketamine (Narketan, Vetoquinol Biovet, Gorzow, Poland; 28 mg/g BW) and placed in a stereotaxic frame (Trent Wells, South Gate, Canada). 6-OHDA hydrobromide (Sigma; 4 mL of a 2 mg/mL solution in 0.9% saline containing 0.02% ascorbic acid) was infused at a rate of 1 mL/min over 4 min into the right medial forebrain bundle at the following coordinates: 4 mm anterior to lambda, 1.3 mm lateral from the midline, and 7.3 mm ventral from the dura surface according to the stereotaxic atlas [24]. The cannula was left at the injection site for an additional 2 minutes post-injection before it was slowly retracted. After the surgery, the animals were left for 2 weeks to allow time for dopamine hypersensitivity to develop.

### Ovariectomy and E2 implants

The animals were anesthetized by isoflurane inhalation and ovariectomized through a dorsolateral skin incision bilaterally. Induction of anesthesia was performed in a chamber with 4% mixture of isoflurane with oxygen, afterward a face mask with 2.5% isoflurane flow was attached for the procedure. After removal of the ovaries, the muscle layer was suture-closed and the skin incision closed with sutures and additional surgical staples (Aesculap, Tuttlingen, Germany). Animals were subcutaneously implanted with silastic implants at the nape of the neck. The implants were made from silastic laboratory tubing (1.47 mm × 1.96 mm, Dow Corning Corporation, USA). The tubing was cut into 1 cm lengths and closed on one end using medical silicone adhesive (A-100, Factor II, Inc., USA). The implants were then filled with 5 mg of E2 benzoate (17-b- E2 3-benzoate; Merck, Darmstadt, Germany) and then closed. Before implantation, the implants were left overnight in sterile physiological saline to allow the initial surge of high E2 levels to be released before use.

### Levodopa and physiological saline injections

Fourteen days after silastic tubing implantation and 6-OHDA stereotactic injection, we started administering daily injections of either L-dopa with benserazide or saline for 2 weeks. We used 4.77 mg/kg BW dose of L-dopa (3,4-Dihydroxy-L-phenylalanine, D-009, Research Biochemical International), while benserazide was administered at 15 mg/kg BW, both were diluted in physiological saline and injected in dose of 2 ml/kg BW. Benserazide is a peripherally acting aromatic L-amino acid decarboxylase or DOPA decarboxylase inhibitor, inhibiting an enzyme that turns L-dopa into dopamine. Since benserazide does not cross the blood–brain barrier, the conversion of L-dopa to dopamine remains limited to brain.

### Chromogenic immunohistochemistry

The primary antibodies used were mouse monoclonal anti-PRL primary antibody; clone 6F11; tested in Western blot, immunohistochemistry and enzyme-linked immunosorbent assay (ELISA); immunohistochemistry performed by Inceboz et al. [25] (Thermo Fisher Scientific; Rockford, IL, USA; Cat# MA1-10597), rabbit polyclonal anti-Syt IV primary antibody; specificity tested and described previously in Ibata et al. [26] (Immuno-Biological Laboratories, Gunma, Japan; Cat# 18977); and mouse monoclonal anti-tyrosine hydroxylase (TH) primary antibody (Sigma Aldrich; St. Louis, MO, USA; Cat# T2928). Chromogenic immunohistochemistry to reveal PRL immunoreactive (PRL-ir) and Syt IV immunoreactive (Syt IV-ir) cells and the amount of lesion made by 6-OHDA using TH staining was performed separately on consecutive tissue slices using avidin/biotin horseradish peroxidase system (Vectastain Elite ABC HRP kit, Vector Laboratories, Burlingame, CA, USA). Briefly, cryosections were fixed in cold methanol with hydrogen peroxide (−20°C) for 15 minutes. After washing the sections in 50 mM KPBS (pH = 7.2), they were incubated in blocking buffer containing either 4% normal horse serum (for determining PRL and TH immunoreactivity) or 4% normal goat serum (for determining Syt IV immunoreactivity), 1% bovine serum albumin, 0.1% Triton X-100 in KPBS at room temperature for 1 hour. The sections were then incubated with either anti-PRL or anti-Syt IV primary antibody (dilution 1:300 and 1:75, respectively) for 1.5 hours at room temperature. After rinses in KPBS, the sections were incubated with either horse anti-mouse biotinylated secondary antibody (for PRL-ir and TH-ir cells; Vector Laboratories; Cat# BA2001) or goat anti-rabbit biotinylated secondary antibody (for Syt IV-ir cells; Vector Laboratories; Cat# BA1000) for 1.5 hour at room temperature. Both secondary antibodies were diluted at 1:500. After adding the ABC-HRP system, the staining was visualized using HRP substrate 3,3’-diaminobenzidine tetrahydrochloride (DAB, Sigma, Merck KGaA, Darmstadt, Germany). Omission of the primary antibodies served as negative control. All sections were immunolabeled simultaneously to ensure the same conditions, such as using identical DAB staining incubation times. Sections were then dehydrated in ethanol series and cleared in xylene. Processed sections were mounted with DPX mountant for histology (Sigma), coverslipped and analyzed under a light microscope (Olympus IX81, Olympus, Tokyo, Japan) with an attached digital camera Olympus IX DP71.

### Double immunofluorescence

Following fixation in methanol and incubation in blocking buffer with a mixture of 4% normal goat serum and 4% normal donkey serum, the sections for double immunofluorescence were incubated in a mixture of both primary antibodies; anti-PRL antibody (1:300) and anti-Syt IV antibody (1:75). The primary antibodies were detected using Alexa Fluor conjugated secondary antibodies AF 488 goat anti-rabbit and AF 555 donkey anti-mouse (Invitrogen, USA; Cat# A11008 and A31570, respectively). After incubation, slices were immersed in 0.1% Sudan Black (Sigma) in 70% vol/vol ethanol for 5 minutes to suppress lipofuscin background autofluorescence. Finally, sections were mounted using Vectashield antifade mounting medium with DAPI for DNA labeling (Vector Laboratories; Cat# H1200) and coverslipped. Slices were analyzed using AxioImager.Z1 with Apotome attachment for optical sectioning (Carl Zeiss MicroImaging, Inc., Heidelberg, Germany; 63× oil immersion lens, NA 1.4, image size 1388 × 1040 pixels, 14.435 mm^2^, pixel size 0.1 mm).

### Semi-quantification of immunohistochemical staining

Chromogenic immunohistochemistry signals of PRL and Syt IV were quantified densitometrically with MCID, M4 image analyzer (Imaging Research Inc., St. Catharines, Ontario, Canada) in the adenohypophysis region of the pituitary gland sections. The relative optical density (ROD) measurements were performed on six sections for each animal. With every new slide measurement, the ROD of the coverslip with glass slide and DePeX was subtracted from the picture as the background. For examining the extent of dopaminergic lesions using TH staining, we analyzed three consecutive brain slices in the region of SN. On each brain slice, we outlined an area of the same size in SN and in the cortex, which represented the background. ROD measurements are presented as a percentage of destroyed dopaminergic neurons compared to the control hemisphere.

### Proportion of Syt IV-PRL-expressing neurons in adenohypophysis

Images from immunofluorescent double staining procedure were used to identify the proportion of ir-PRL cells that were also positive for Syt IV (90 images in total were analyzed; six images per animal). Cell counting was performed using Fiji (ImageJ) plug in cell counter. An individual cell was determined as PRL or Syt IV positive, if there was an immunoreactive signal at least partly enclosing the cell nuclei stained with DAPI. Altogether, 6549 PRL-ir cells were examined for Syt IV immunoreactivity.

### Serum PRL and E2 levels

Serum PRL levels were measured using rat PRL ELISA kit with the detection limit of 0.8 mg/L (Cusabio, Wuhan Huamei Biotech Co., Ltd., China) following instructions from the manufacturer. Serum E2 levels were measured using automated electrochemiluminescence immunoassay Elecsys E2 III (Roche Diagnostics International Ltd., Switzerland) with a detection limit of 18 pmol/L performed on Cobas e411 analyzer (Roche Diagnostics, Mannheim, Germany).

### Morphometry of lactotroph nuclei and estimates of pituitary size

We analyzed the sizes of PRL-ir cell nuclei with Fiji (ImageJ). Using images obtained from double immunofluorescent staining of Syt IV-ir and PRL-ir cells with DAPI, we measured diameters of 120 PRL-ir cells per animal. The data are presented as means ± standard error (SE). Size of pituitary glands was estimated from maximum cross-sectional areas of serial coronal sections of the whole gland, which were photographed with Olympus DP71 digital camera connected to microscope Olympus IX81 (Olympus, Tokyo, Japan) and measured using Fiji (ImageJ).

### Statistical analysis

Statistical analysis was performed using SPSS 25.0 (IBM, Armonk, NY, USA). All data were tested for normality and homogeneity of variance using the Kolmogorov-Smirnov, Shapiro-Wilk, and Levene’s tests and were found to be normally distributed. Significant differences between treatment groups were assessed with one-way analysis of variance (ANOVA) with planned contrasts. Data are presented as mean ± SE. *p* < 0.05 was considered significant.

## RESULTS

This section should provide a concise and precise description of the experimental results. It may be divided by subheadings as illustrated below.

### Dopaminergic lesions

The extent of dopaminergic lesions did not differ between the 6-OHDA groups E2 and E2 + L-dopa (89.2 ± 6.84% and 87.4 ± 5.84%, respectively). In the intact group, there were no lesions in the area of SN.

### Silastic tubing 17β- E2 implants resulted in high serum levels of E2

**FIGURE 1:**
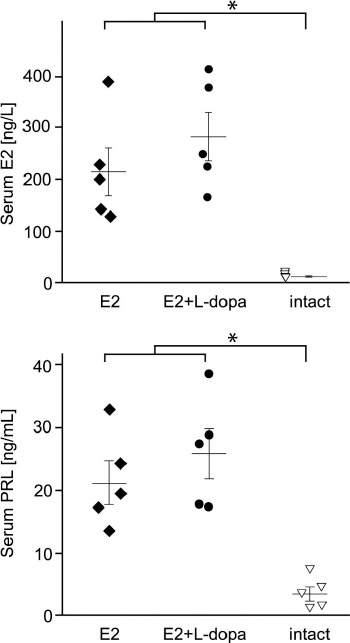
Serum concentrations of E2 and PRL. In both cases, the concentrations are significantly higher in treatment groups that received E2 (N = 10) compared to healthy animals (N = 5). There were no significant differences between treatment groups E2 (N = 5) and E2+L-dopa (N = 5) in either E2 or PRL serum concentrations. Differences between treatment groups were assessed using one-way ANOVA with planned contrasts. An asterisk denotes a statistically significant difference (p < .05). Error bars indicate standard errors. Individual symbols represent measurements from individual animals in the respective treatment groups. E2 - 17b-estradiol; PRL – prolactin; L-dopa – levodopa.

Levels of E2 were measured from serum collected from euthanized animals. After 28 days, the serum levels produced by the silastic implants containing 5 mg of E2 were 221.5 ± 46.5 pg/ml (*n* = 5) in treatment group E2 and 289.8 ± 47.2 pg/ml (*n* = 5) in treatment group E2 + L-dopa. The average serum level of E2 in intact animals was 17.5 ± 1.3 pg/ml (*n* = 5) (Figure 1).

### Increased serum levels of PRL

Serum PRL was significantly increased in treatment groups that received E2 compared to intact animals, *t* (12) = 2.61, *p* < 0.05 (2-tailed), *r* = 0.60, while L-dopa had no significant additional effect on serum PRL concentrations (Figure 1).

### Pituitary size

**FIGURE 2:**
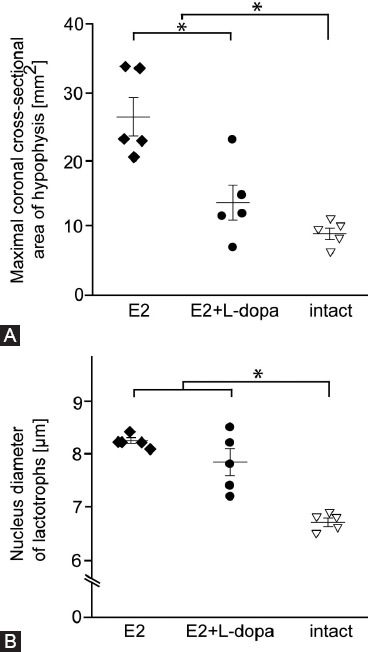
The effect of L-dopa on morphometric parameters of pituitary glands. (A) Differences in the size of pituitary glands. Pituitary glands were significantly larger in treatment groups that received E2 (N = 10) compared to healthy animals (N = 5). Treatment group E2 + L-dopa (N = 5) shows a significant reduction in the size of pituitary glands compared to treatment group E2 (N = 5). (B) Nucleus diameter of lactotrophs. The nuclei of lactotrophs were significantly larger in treatment groups that received E2 (N = 10) compared to healthy animals (N = 5). Treatment with L-dopa did not have a significant effect on the size of lactotroph nuclei. Differences between treatment groups were assessed using one-way ANOVA with planned contrasts. An asterisk denotes a statistically significant difference (p < .05). Error bars indicate standard errors. Individual symbols represent measurements from individual animals in the respective treatment groups. E2 - 17b-estradiol; L-dopa – levodopa.

Planned contrasts revealed that high concentrations of E2 used in our study lead to significant increases in sizes of pituitary glands compared to intact animals, *t*(12) = 3.96, *p* < 0.05 (2-tailed), r = 0.75. Treatment with L-dopa resulted in significant reduction of pituitary glands, t(12) = 3.96, *p* < 0.05 (2-tailed), r = 0.75 (Figure 2A).

### 17β- E2-induced adenomas show enlarged lactotroph nuclei

Microscopic examination of pituitary tissue revealed enlarged nuclei of PRL-ir cells in pituitary adenomas compared to pituitary glands from healthy intact animals (Figure 2B). Planned contrasts ANOVA showed that nuclei of lactotrophs in pituitary adenomas were significantly larger in size than nuclei of lactotrophs in healthy animals, *t*(12) = 7.14, *p* < 0.05 (2-tailed), r = 0.90. While treatment with L-dopa had no statistically significant effect (*p* *=* 0.08), its influence on the size of the nuclei is still considerable, since the calculated effect size (r) of .51 indicates a statistically large effect (Figure 2B).

### Expression of PRL in 17β- E2-induced pituitary adenomas

**FIGURE 3:**
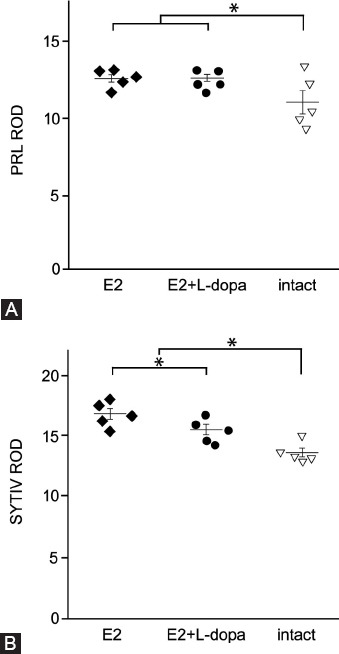
The effect of L-dopa on the expression of PRL and Syt-IV. (A) Densitometric quantification of PRL immunosignal shows that the expression of PRL was significantly higher in treatment groups that received E2 (N = 10) compared to healthy animals (N = 5). Treatment with L-dopa did not have a significant effect on the expression of PRL. (B) Densitometric quantification of Syt-IV immunosignal shows that the expression of Syt-IV was significantly higher in treatment groups that received E2 (N = 10) compared to healthy animals (N = 5). Treatment group E2 + L-dopa (N = 5) shows significant reduction in the Szt IV expression compared to treatment group E2 (N = 5). Differences between treatment groups were assessed using one-way ANOVA with planned contrasts. An asterisk denotes a statistically significant difference (p < .05). Error bars indicate standard errors. Individual symbols represent measurements from individual animals in the respective treatment groups. E2 - 17b-estradiol; PRL – prolactin; SYT IV – synaptotagmin IV; L-dopa – levodopa; ROD - relative optical density.

**FIGURE 4:**
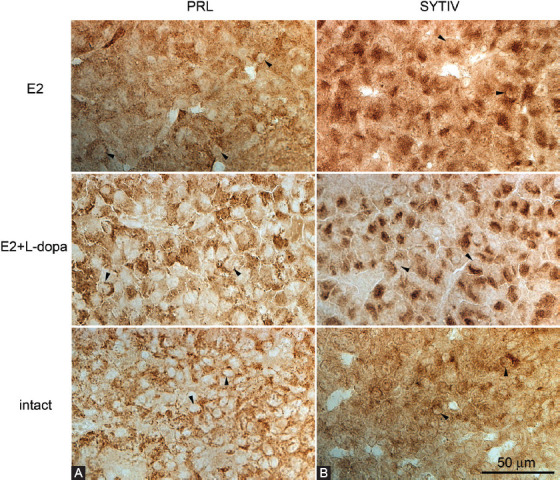
Chromogenic immunolabeling of PRL and Syt IV. Chromogenic immunolabeling in the adenohypophysis of E2-induced pituitary adenomas of rats from treatment groups E2 and E2+L-dopa and from healthy pituitary glands from intact animals (magnification 400x). Arrowheads indicate cell nuclei. Brown staining indicates the cells with positive expression. E2 - 17b-estradiol; PRL – prolactin; SYT IV – synaptotagmin IV; L-dopa – levodopa.

The levels of PRL in tissue of pituitary glands were investigated by semi-quantitative densitometry of immunohistochemical signals. Analysis of relative PRL immunostaining intensity using planned contrasts ANOVA showed significant increase in both treatments compared to intact healthy animals (*t*(12) = 2.65, *p* < 0.05 (2-tailed), r = 0.61), but no significant difference between the two treatments (*t*(12) = −0.06, *p* = 0.96 (2-tailed), *r* = 0.03) (Figures 3A and 4).

### Expression of Syt IV in E2-induced pituitary adenomas

**FIGURE 5:**
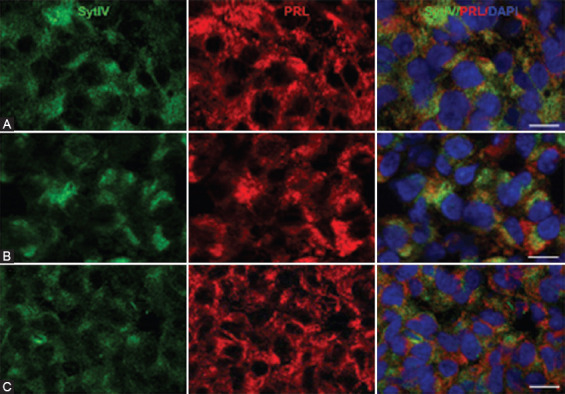
Double immunofluorescence labeling of Syt IV and PRL. Nuclei were counterstained with DAPI. (A) Adenohypophysis of rats from treatment group E2. (B) Adenohypophysis of rats from treatment group E2+L-dopa. (C) Adenohypophysis of intact control animals. Scale bars: 10 µm. E2 - 17b-estradiol; PRL – prolactin; SYT IV – synaptotagmin IV; L-dopa – levodopa.

The levels of Syt IV in tissue of pituitary glands were investigated by semi-quantitative densitometry of immunohistochemical signals. Relative Syt IV immunostaining intensity (Figures 3B and 4) showed that expression of Syt IV was significantly higher in treatment groups that received E2 compared to intact animals, *t*(12) = −4.99, *p* < 0.05 (2-tailed), r = 0.82, namely 16.8 ± 0.9 (*n* = 5) in treatment group E2 and 15.5 ± 0.9 (*n* = 5) in treatment group E2 + L-dopa. L-dopa injections resulted in a small but statistically significant decrease of Syt IV immunostaining intensity, *t*(12) = 2.22, *p* < 0.05 (2-tailed), r = 0.66 (Figures 4B and 5).

### Syt IV is expressed in most lactotrophs

Cellular localization of Syt IV in the adenohypophysis of pituitary adenomas and pituitary glands of intact animals was investigated using double immunofluorescence labeling of Syt IV and PRL (Figure 5). Syt IV was expressed in most PRL-positive cells in treatment groups E2 (93.7 ± 4.9%) and E2 + L-dopa (91.7 ± 2.1%) and also in intact animals (96.7 ± 1.4).

## DISCUSSION

Hemiparkinsonian ovariectomized adult female rats with unilateral lesions of dopaminergic nigrostriatal neurons, reflecting premature natural/iatrogenic menopause were used in our experiment to investigate the effects of levodopa and high doses of E2 on histological parameters and secretory ability of pituitary lactotrophs and changes in their expression of Syt IV. In brief, our data show for the first time that high levels of serum E2 result in upregulation of Syt IV in PRL-ir cells of adenohypophysis. L-dopa, the first line of treatment for PD results in a decrease in expression of Syt IV.

Physiological levels of estrogen in gonadally intact female rats normally fluctuate during the 4-5 day estrous cycle from 17 to 88 pg/ml [18] but were reported in some cases to reach close to 100 pg/ml [21] or even 200 pg/ml [27] during proestrus surge. Silastic tubing implants used in this study were designed to produce approximately 75-100 pg/ml of serum E2, however, there seems to be widespread variability [28-30]. One study showed that 5 mg of E2 in silastic tubing implants can result in as high as 218 pg/ml of plasma E2 levels [21]. Long-term treatment with pharmacological doses of estrogens induces PRL-secreting pituitary tumors in rodents [9]. E2 stimulation induces gradual hypertrophy and hyperplasia of lactotrophs and leads to vascular channel enlargement [10,31]. In Fischer-344 female rats, maintenance of constant elevated systemic E2 level within the range of 100-300 pg/ml induces prolactinomas very rapidly [11]. Duration of E2 administration also has to be taken into account since longer exposure results in a higher proliferation rate of lactotrophs and expression of factors involved in the genesis of pituitary tumors [31]. In our study, the serum levels of E2 in rats with implants were in the range between 100 and 400 pg/ml, and substantial prolactinomas with enlarged vascular channels developed in both treatment groups receiving E2.

In this study, 5 mg E2 implants lead to a significant increase in the size of pituitary glands and elevated PRL serum levels compared to intact control animals, but the amount of serum PRL measured was still five to ten-fold lower than the levels reported in studies that used E2 benzoate silastic tubing implants or other methods to experimentally produce prolactinomas. For example, in one previous study, after 20 days, serum PRL levels reached 231 ng/ml [32]. In Xu et al. [33], the PRL serum levels reached as high as 1262 ng/ml plasma PRL after 30 days. It has to be noted, however, that the implants in both studies contained 10 mg of E2.

The increase in the size of the pituitary in our study was 2-3.5-fold, which is similar to data from previous studies [33]. Along with hypertrophy and hyperplasia of lactotrophs in E2-treated animals, there was a significant increase in the size of their nuclei, which was previously shown in a study on the effects of E2 treatment on the morphology of PRL-ir cells in female middle-aged rats [34]. Enlargement of vascular channels was also observed, especially in the treatment group E2, corroborating findings by previous studies [32]. Treatment with L-dopa significantly decreased the effect of E2 on the size of prolactinomas as well as the size of vascular channels and showed a statistically large effect on the size of the nuclei.

PRL expression in lactrotrophs was elevated significantly in animals that received E2 treatment compared to intact animals. E2 affects the secretion of PRL at two levels; directly at the lactotroph level and within the hypothalamus, where it modifies the activity of the neuroendocrine neurons known to control PRL secretion [8]. At the lactotroph level, E2 controls PRL gene expression by binding to estrogen receptors (ER) and through orchestrated events involving several different growth factors, such as transforming growth factor b [11]. E2 reduces the expression of lactotroph dopamine receptors, and long-term estrogen treatment reduces levels of TH and dopamine content in tuberoinfundibular dopaminergic neurons that are considered the major source of dopamine in the anterior pituitary gland. The adenohypophysis of adult female rats expresses ERs, such as ERa, ERb, and also the truncated ER product-1, TERP-1 [35]. ERa is prevalent and localized mainly in lactotrophs. Interestingly, it has been shown that estrogen treatment of ovariectomized rats does not regulate ERa transcription and slightly decreases ERb mRNA levels but dramatically induces TERP-1 mRNA [35]. Within the rat pituitary, E2 controls PRL gene expression by direct binding of activated ERs to specific DNA sequences located in the promoter of the rat PRL gene [8].

Dopaminergic inhibition of PRL synthesis and release is mediated through D2 dopamine receptors. The inhibitory response of PRL secretion has been shown to be coincident with inhibition of adenylate cyclase activity, a characteristic response of D2 receptor activation involving G-protein [36,37]. Interestingly, treatment with L-dopa did not result in lower serum PRL or lower expression of PRL in the adenohypophysis, despite smaller prolactinomas. Estrogens are known to modify the response of the rat lactotroph to physiological inhibitors such as dopamine; dopamine is less potent as an inhibitor of PRL secretion when lactotrophs are exposed to E2 [38,39]. E2 may exert this effect by decreasing the number of D2 dopamine receptors [40,41], which could explain no significant effect of L-dopa on either serum PRL or expression of PRL. No apparent effect of L-dopa on serum PRL may also be explained by the fact that the lowering of serum PRL concentrations by L-dopa is only transient [42]. As well as directly acting on the pituitary gland and inhibiting PRL release, L-dopa causes a decrease in serum levels of PRL by stimulating the uptake of this hormone in the periphery, an effect which only lasts for several hours after L-dopa injection, after which a rapid restoration of serum PRL levels occurs without a substantial release from the pituitary. The inconsistencies between dopaminergic activity and serum PRL levels have also been attributed to other hypothalamic factors that may alter PRL release, such as GABA, somatostatin, and calcitonin [43]. Nevertheless, L-dopa did have a significant effect on the size of pituitary glands by inhibiting lactotroph proliferation [9]. The size of prolactinomas in L-dopa treated animals was significantly smaller since dopamine inhibits E2-stimulated lactotroph proliferation and their basally high-secretory tone [9,12].

A previous study [44] found that fluctuations of E2 during the estrous cycle did not affect the expression of Syt IV in rat hippocampus, however, we show that expression of Syt IV in PRL-ir cells was upregulated in all animals receiving E2 compared to intact animals; we postulate that Syt IV is an estrogen-responsive immediate early gene in the pituitary gland. Its expression may be mediated directly through ERs and their binding to the DNA promoter regions of the Syt IV gene. Syt IV stands out as an anomaly among other synaptotagmins, which act as Ca^2+^ sensors in regulated exocytosis and neurotransmitter release. Because of Ser substitution in the domain that inactivates Ca^2+^/phospholipid binding activity and SNARE binding activity *in vitro* [23,45], Syt IV has most often been regarded as a negative regulator of Ca^2+^-dependent exocytosis. However, positive roles of Syt IV in Ca^2+^-dependent exocytosis have also been reported [13]. Since we found that the addition of L-dopa significantly decreased the expression of Syt IV, as well as decreasing the size of prolactinomas, we may hypothesize that in our case Syt IV expression may be positively linked to PRL exocytosis. L-dopa acted through lactotroph D2 receptors through inhibition of adenylate cyclase on the expression of immediate early gene Syt IV, which was shown to be coexpressed with PRL in over 90% of lactotrophs. Further investigations into exact mechanisms are needed to elucidate E2 and L-dopa effect on Syt IV expression and involvement of Syt IV in PRL secretion.

PD is to this day a chronic incurable disease, so treating its symptoms is the best way to improve patients’ quality of life. One important aspect is the management of an array of different non-motor symptoms. In our study, middle-aged rats with ovariectomy represent female patients in their postmenopausal stage of life. This is similar to human PD patients since PD is generally a disease of the elderly and its incidence increases with age. PRL is known to play an important role in the modulation of stress response and emotion regulation [46]. Since estrogen and dopamine are major opposing factors affecting PRL secretion, postmenopausal female PD patients on hormone replacement therapy should be closely monitored for PRL levels. On the one hand, a high level of estrogens may lead to the formation of prolactinomas [47], and on the other hand, PD treatment with L-dopa and other antiparkinsonian drugs such as cabergoline suppress PRL secretion from the pituitary gland and may lead to hypoprolactinemia. Isolated hypoprolactinemia is an extremely rare phenomenon (mostly of iatrogenic cause) and therefore not much is known about it. It is associated with sexual dysfunction and depression [48]. This could be particularly dangerous for PD patients since symptoms could be attributed to non-motor symptoms of PD and easily overlooked.
